# Plugging the Holes in Water Distribution Systems: Deficiencies May Contribute to Gastrointestinal Illnesses

**DOI:** 10.1289/ehp.122-A195

**Published:** 2014-07-01

**Authors:** Julia R. Barrett

**Affiliations:** Julia R. Barrett, MS, ELS, a Madison, WI–based science writer and editor, has written for *EHP* since 1996. She is a member of the National Association of Science Writers and the Board of Editors in the Life Sciences.

Deficiencies in drinking water distribution systems can result in illness, even if the water meets health standards as it leaves a treatment facility.^1^ A new review in *EHP* discusses the routine malfunctions in distribution networks that contribute to background levels of gastrointestinal illness and highlights critical research needs.^2^

The most common adverse health effects from unsafe drinking water are diarrheal illnesses, which accounted for nearly 88% of the identified outbreaks of drinking water–associated illness in the United States from 1971 to 2006.^1^ This figure likely represents only a small percentage of the actual incidence, however, since these illnesses often aren’t reported to doctors.^3^

**Figure:**
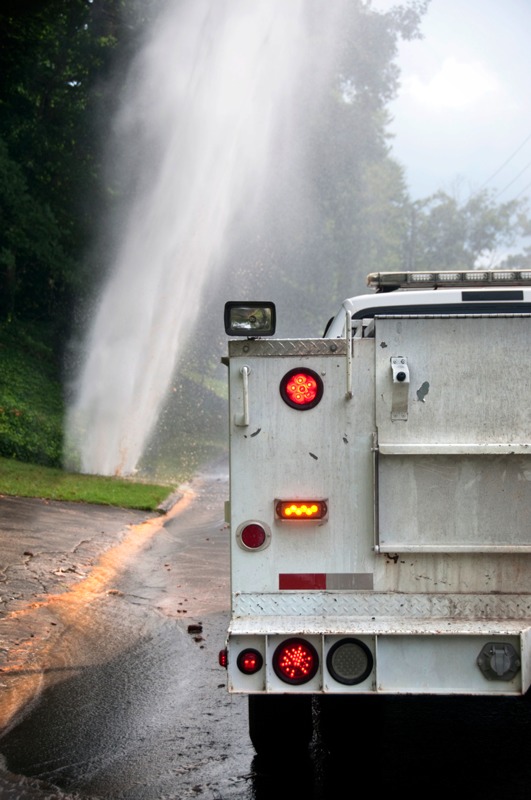
Pipe breaks and loss of water pressure provide opportunities for treated drinking water to become contaminated en route to consumers. © Eneri LLC/GettyImages

Measuring exposure to pathogens in water distribution systems is challenging because water is not continuously monitored between the treatment plant and the tap.^4^ Typical testing methods do not capture factors such as transient fluctuations in water pressure, which can dredge up sediments, dislodge pathogens from biofilms within the pipes, and allow adjacent soil water to enter the system.

Furthermore, chlorine residual (i.e., the amount of chlorine disinfectant left in the water after initial application) is sampled only at certain points within a system, typically once per day. “Even if a system is in compliance, it may experience periods or certain locations where residual levels are less than what’s required to prevent recontamination,” says Julie Shortridge, a doctoral candidate in the Department of Geography and Environmental Engineering at The Johns Hopkins University, who was not involved with the review.

In developed countries, aging distribution systems are increasingly prone to pipe breaks and consequently diminished water pressure. In developing countries, systems may be overburdened and inadequately maintained, leading to breaches in pipe integrity and low water pressure. These systems also may be intermittently operated, with nonpressurized periods between supply cycles.

“Our review revealed there are a relatively small number of studies specifically designed to investigate water distribution systems as a risk factor for gastrointestinal illness,” says lead author Ayse Ercumen, a postdoctoral researcher in the Division of Epidemiology at the University of California, Berkeley. The studies that do exist suggest recontamination of drinking water once it leaves the treatment plant can increase the risk of gastrointestinal illness, although the literature varies considerably in terms of type of study, distribution system performance, whether participants knew of their potential exposure, and other variables.

The authors analyzed four different sets of studies according to specific research questions. The first set compared gastrointestinal illness among people drinking centrally treated water versus those drinking water treated at the point of use. Another assessed studies of illness in association with pipe damage. A third assessed illness in association with water outages. Finally, a fourth set assessed problems related to disinfection.

Among other findings, the authors concluded that centrally treated tap water was more likely to be associated with gastrointestinal illness than water treated at the point of use. But upon further analysis they found this association was only positive for nonblinded studies—that is, those in which participants knew which type of water they were drinking. They also found that both temporary water outages in otherwise continuous systems and chronic outages in intermittently operated systems were associated with risk of illness.

The authors suggest that future studies could include both randomized controlled trials (for instance, comparing people whose water is treated centrally with those who treat at point of use) and prospective cohort studies. Shortridge agrees that randomized control trials would rigorously evaluate the issue on a small scale, but adds that novel monitoring methods, like the Google search data used in a study she coauthored,^3^ “could be used in tandem with focused control studies aimed at generating insights across different types of systems.”

However, Elena Naumova, a professor at the Tufts University School of Civil and Environmental Engineering, believes research time and dollars would be best focused on strengthening routine monitoring at the municipal level for both infrastructure integrity and surveillance of waterborne illness. Pointing to her own experience,^5^ she says, “Integrating reliable data from multiple sources could lead to tailored strategies for improving both water infrastructure and measurable health outcomes.”
